# Changes in newly notified cases and control of tuberculosis in China: time-series analysis of surveillance data

**DOI:** 10.1186/s40249-021-00806-7

**Published:** 2021-02-24

**Authors:** Ye-Sheng Wang, Wen-Long Zhu, Tao Li, Wei Chen, Wei-Bing Wang

**Affiliations:** 1 Department of Epidemiology, School of Public Health, Fudan University, No. 130 Dong’an Road, Xuhui District, Shanghai, China; 2 Key Laboratory of Public Health Safety of Ministry of Education, Fudan University, Shanghai, China; 3 National Center for Tuberculosis Control and Prevention, Chinese Center for Disease Control and Prevention, Beijing, China

**Keywords:** Tuberculosis, Trend, Control, Regions, China

## Abstract

**Background:**

China has made progress in tuberculosis control, but this disease remains a burden in many regions of China. We performed time-series analysis to examine changes in the rates of newly notified and newly smear-positive cases of tuberculosis in different regions of China from 1997 to 2018 and assessed the effect of the current control program.

**Methods:**

National and provincial notification data on tuberculosis from 1997 to 2018, which covers 31 provinces in the mainland of China, were extracted from the Chinese public health science data center. The annual percentage changes in newly notified and smear-positive cases were analyzed using a joinpoint regression method.

**Results:**

There were 18 646 672 newly notified tuberculosis cases from 1997 to 2018, with the greatest number in 2005. A total of 6 605 414 of these cases (35.42%) were smear-positive cases. The number of newly notified cases in China overall decreased (96.88–59.27 cases per 100 000) significantly during the most recent years. The decline during this period ranged from −3.9% (95% *CI* −5.7 to −2.9) in the western region to −4.3% (95% *CI* −4.8 to −3.7) in the eastern region. Most provinces had significant declines in newly notified and smear-positive cases, whereas the decline of newly smear-positive cases in Xinjiang was about half of that observed during the same period in China overall (−4.1% vs −9.9%). In addition to disparities in annual percentage changes, the rate of newly notified cases was higher in the western region than in the eastern and central regions.

**Conclusions:**

The burden of tuberculosis has been on declining throughout China during recent years, but tuberculosis in western China continues to be a public health emergency that needs to be urgently addressed. Effective prevention and control strategies are needed for regions with high disease burdens and those with increasing or unchanging numbers of newly notified and smear-positive cases of tuberculosis.
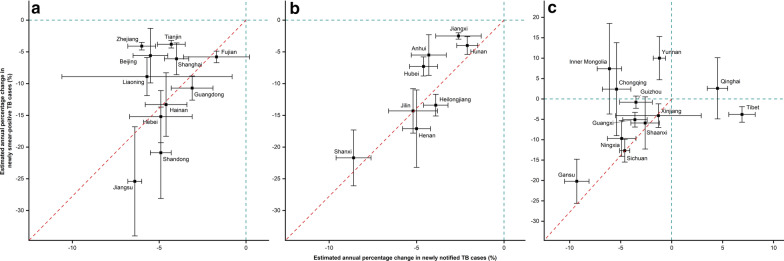

## Background

Tuberculosis (TB) is the leading cause of death from infectious pathogens worldwide, and was responsible for approximately 1.45 million deaths worldwide during 2018 [1]. About one-quarter of the population worldwide, an estimated 1.7 billion people, are infected with the causative bacterium (*Mycobacterium tuberculosis*), and have active disease or latent/asymptomatic disease [2]. Although TB disproportionately affects middle- and low-income countries, economically developed regions are also vulnerable [3]. Thus, development of interventions against the TB epidemic is an urgent global public health priority [4].

In an effort to end the TB epidemic within the next generation, the first ever United Nations High Level Meeting (UNHLM) on TB convened during 2018. At that time, world leaders committed to a series of measures to end TB, which included successfully treating 40 million people with TB and providing TB preventive treatment to 30 million other people between 2018 and 2022 [5]. The annual global decline in the incidence of TB was about 2.0% from 2017 to 2018, higher than the annual declines during the previous 18 years [1]. However, as the global community looks to meet ambitious targets for reduction and even elimination of TB by 2050 [6], the annual declines in incidence must be much greater [7]. Therefore, more interventions with proven efficacies and investments in new strategies are needed to achieve the UNHLM targets and to eliminate TB by 2030.

TB is a notifiable disease in China and TB control has been a part of China’s public health program since the 1950s. During the past two decades, China implemented a nationwide scale-up of its TB control program to address the growing burden of TB. This program is based on use of directly observed therapy short-course (DOTS), and was implemented in 13 provinces of China during the 1990s, and expanded nationwide after 2000. The 2010 national TB prevalence survey in China indicated that scale-up of the DOTS strategy led to a 65% decline in the prevalence of smear-positive TB and a 48% decline of bacteriologically confirmed TB between 1990 and 2010 [8]. However, there are disparities in the declines reported for the eastern, central, and western regions of China [9]. The smallest declines were in the western provinces, where the prevalence of TB is greatest [10].

There have been no recent systematic data published on changes of disease burden and regional disparities of TB in China, except for some studies which reported overall incidence trends [11, 12]. The current study aims to examine changes in newly notified and newly smear-positive cases of TB in different regions of China based on national routine surveillance data from 1997 to 2018, and to assess the effectiveness of the TB control program in China.

## Methods

### Data collection

The Chinese government established a routine reporting system for selected infectious diseases during the 1950s, and data are available for 31 provinces in the mainland of China (about 1.4 billion people). This system was web-based since 2003 and operates through administrative grading responsibility and territorial management [13]. Hong Kong, Macau, and Taiwan were not included in the present analysis because of a lack of data availability.

The newly notified TB cases examined in this study were extracted from the notifiable infectious disease report database, which is open and available from the public health and science data center of the Chinese Center for Disease Control and Prevention [14]. These data include the number of newly notified TB cases, and patient status as sputum smear-positive, sputum smear-negative, sputum not done, and sputum culture-positive TB. Due to the high risk of TB transmission, patients with smear-positive TB receive the greatest attention. Therefore, in addition to considering newly notified TB cases, we also examined newly smear-positive TB cases from 1997 to 2018 at the provincial level. In addition, we have contacted with National Center for Tuberculosis Control and Prevention to verify the data before analysis. According to the discussion, the annual data were generally correct and consistent with the data reported to the World Health Organization (WHO).

To explain factors attributed to disparities in disease burden in different provinces, four social-economic variables were described: (a) economic levels: gross domestic product per capita (PGDP); (b) demographic characteristic: population density (persons per square kilometers; PD); (c) accessibility to and coverage of health facilities: including number of beds in medical institutions (NBMI) and number of medical personnel (NMW). The health service data were collected from the China Health Statistical Yearbook [15], and other socio-economic variables were obtained from the China Statistical Yearbook [16].

### Statistical analysis

The provinces were divided into three regions (eastern, central, and western regions), as defined by the National Bureau of Statistics of China and according to sociodemographic status. The eastern region included Beijing, Fujian, Guangdong, Hainan, Hebei, Jiangsu, Liaoning, Shandong, Shanghai, Tianjin, and Zhejiang; the central region included Anhui, Heilongjiang, Henan, Hunan, Hubei, Jiangxi, Jilin, and Shanxi; and the western region included Chongqing, Gansu, Guangxi, Guizhou, Inner Mongolia, Ningxia, Qinghai, Shaanxi, Sichuan, Tibet, Xinjiang, and Yunnan [17].

Annual notification rate was defined as the number of newly notified cases of TB per 100 000 people. For each province, the numbers of newly notified and smear-positive cases were calculated. For analysis of TB in the eastern, central, and western regions, the numbers of newly notified and smear-positive TB cases from all provinces within each region were calculated. Population data were from the China Statistical Yearbook [16].

Joinpoint regression models were used to analyze the annual percentage changes of newly notified cases and their significance [18]. This analysis, which is often used to describe changes in trends, fits a series of joined linear models of the natural logarithm of annual incidence, using calendar year as an independent variable [19]. In this analysis, the response variable was the natural logarithm of the notification rate, and the independent variable was the year of notification. A maximum of five joinpoints were used for estimation, as suggested by the program developers, and the Bayesian information criterion (BIC) was used to select the model with the best fit. Once a joinpoint was determined, the annual percentage changes and 95% confidence intervals (*CI*s) of each period (segment) was determined. An annual percentage change was considered to be significant when its 95% *CI* did not include zero (*P* < 0.05). In describing trends, the terms “increase” and “decrease” were used when the slope (annual percentage change) was significant. The term “stable” was used to refer to a non-significant change (i.e., a stable notification rate or a notification rate that was unreported or only reported sporadically).

Then, spatial auto-correlation analysis was used to examine the spatial heterogeneity of annual percentage changes during the most recent period (i.e., after the last joinpoint if a joinpoint was identified, or the whole period if a joinpoint was not identified). Auto-correlation statistics are commonly used to examine spatial dependence in geographic data [20]. The spatial auto-correlation has two major features: (*a*) the global spatial auto-correlation, which indicates the overall relationship of all the research units in the area; and (*b*) the local indicators of spatial association (LISA), which indicate the influence of individual locations on the magnitude of the global statistic and the locations and types of clusters [21]. The spatial weights were created using the rook contiguity rule, and applied to describe the spatial relationships among provinces. The spatial distribution of annual percentage changes of notification rates from each province were determined by calculating the global Moran’s *I* and LISA. The following equation was used to calculate the global Moran’s *I* [22]:

$$I = \frac{{n \cdot \sum\nolimits_{i = 1}^{n} {\sum\nolimits_{j = 1}^{n} {w_{ij} \left( {x_{i} - \overline{x} } \right)\left( {x_{j} - \overline{x} } \right)} } }}{{\left( {\sum\nolimits_{i = 1}^{n} {\sum\nolimits_{j = 1}^{n} {w_{ij} } } } \right)\sum\nolimits_{i = 1}^{n} {\left( {x_{i} - \overline{x} } \right)^{2} } }},\;i \ne j$$

The local Moran’s *I* was used to make the LISA cluster maps, and was computed as follows [23]:

$$I_{i} = \frac{{\sum\nolimits_{j = 1}^{n} {w_{ij} \left( {x_{i} - \overline{x} } \right)\left( {x_{j} - \overline{x} } \right)} }}{{\frac{1}{n}\sum\nolimits_{i = 1}^{n} {\left( {x_{i} - \overline{x} } \right)^{2} } }},\;i \ne j$$

where n is the number of provinces, ${x}_{i}$ and ${x}_{j}$ are the annual percentage changes of provinces i and j, $\stackrel{-}{x}$ is the average of the annual percentage changes of all provinces, and ${w}_{ij}$ is the spatial weight matrix corresponding to the provinces pair i and j. The value of Moran’s *I* usually ranges from −1 to 1, with positive values representing a positive spatial correlation and negative values representing a negative spatial correlation.

IBM SPSS version 25 (SPSS Inc., Chicago, IL, USA) was used to compare notification rates in different regions and at different times. Joinpoint regression analysis was performed using the Joinpoint Regression Program version 4.8.0.1 (Statistical Research and Applications Branch, National Cancer Institute). ArcGIS version 10.2 (ESRI, Redlands, CA, USA) was used to perform the spatial auto-correlation analysis and to plot the maps.

## Results

### Changes in newly notified TB cases in three geographic regions

From 1997 to 2018, there were 18 646 672 newly notified TB cases in China, and 35.42% of these cases (6 605 414) were smear-positive (Table 1). The number of newly notified cases among all provinces increased significantly from 1997 to 2005, especially from 2002 to 2005 (43.58–96.88 cases per 100 000). For this period, joinpoint regression analysis indicated an annual percentage change of 28.5% (95% *CI* 15.3–43.1). The annual percentage changes during this period in the eastern, central, and western regions were 29.1% (95% *CI* 13.4–46.8), 36.2% (95% *CI* 23.0–50.8), and 25.0% (95% *CI* 6.8–46.2), respectively.

**Table 1:** Joinpoint analysis of newly notified TB cases in different geographic regions of China

	Newly notified TB cases*	Annual percentage change (95% *CI*)	*P* value
All provinces			
1997 to 2002	34.69 to 43.58	3.5 (0.3–6.7)	** < 0.001**
2002 to 2005	43.58 to 96.88	28.5 (15.3–43.1)	** < 0.001**
2005 to 2018	96.88 to 59.27	−3.8 (−4.3 to −3.3)	** < 0.001**
Eastern provinces			
1997 to 2002	33.23 to 33.13	−0.4 (−3.8–3.1)	0.800
2002 to 2005	33.13 to 72.06	29.1 (13.4–46.8)	** < 0.001**
2005 to 2018	72.06 to 42.81	−4.3 (−4.8 to −3.7)	** < 0.001**
Central provinces			
1997 to 2002	31.03 to 41.04	3.7 (0.6–6.9)	** < 0.001**
2002 to 2005	41.04 to 111.51	36.2 (23.0–50.8)	** < 0.001**
2005 to 2018	111.51 to 59.78	−4.2 (−4.7 to −3.8)	** < 0.001**
Western provinces			
1997 to 2002	40.90 to 55.69	6.0 (1.3–10.9)	** < 0.001**
2002 to 2005	55.69 to 121.13	25.0 (6.8–46.2)	** < 0.001**
2005 to 2018	121.13 to 84.13	−3.9 (−5.7 to −2.9)	** < 0.001**

The bold indicated that the changes were significant

*The number of newly notified TB cases (per 100 000) at the first and the last year of the indicated segment

However, from 2005 to 2018, there was a decrease of newly notified TB cases in China overall (96.88–59.27 cases per 100 000). The joinpoint regression results indicated an annual percentage change of −3.8% (95% *CI* −4.3 to −3.3). The decline during this period ranged from −3.9% (95% *CI* −5.7 to −2.9) in the western region to −4.3% (95% *CI* −4.8 to −3.7) in the eastern region.

The numbers of newly smear-positive TB cases had similar trends (Table 2). The overall number of newly smear-positive cases (all provinces) increased from 11.21 per 100 000 in 1997 to 41.90 per 100 000 in 2005, and then decreased to 16.23 per 100 000 in 2018. However, the decline varied among different time periods, and changed markedly after 2010. In particular, the annual percentage change was −3.0% (95% *CI* −6.6 to 0.8) from 2005 to 2010 and −9.9% (95% *CI* −12.7 to −6.9) from 2010 to 2018. Notably, the changes for the period of 2005–2010 were not significant in the central and western regions, but the eastern region had a significant decline during that period (−2.9%, 95% *CI* −5.5 to −0.1).

**Table 2:** Joinpoint analysis of newly smear-positive TB in different geographic regions of China

	Newly smear-positive TB*	Annual percentage change (95% *CI*)	*P* value
All provinces			
1997 to 2002	11.21 to 12.49	1.1 (−3.5 to 5.8)	0.600
2002 to 2005	12.49 to 41.90	42.8 (22.5–66.5)	** < 0.001**
2005 to 2010	41.90 to 34.21	−3.0 (−6.6 to 0.8)	0.100
2010 to 2018	34.21 to 16.23	−9.9 (−12.7 to −6.9)	** < 0.001**
Eastern provinces			
1997 to 2002	13.07 to 13.67	1.0 (−1.9 to 4.1)	0.500
2002 to 2005	13.67 to 34.10	29.3 (16.3–43.8)	** < 0.001**
2005 to 2010	34.10 to 29.54	−2.9 (−5.5 to −0.1)	** < 0.001**
2010 to 2018	29.54 to 12.61	− 10.3 (− 11.5 to − 9.2)	** < 0.001**
Central provinces			
1997 to 2002	11.36 to 12.49	− 0.1 (− 4.9 to 4.8)	1.000
2002 to 2005	12.49 to 51.43	54.7 (32.5–80.7)	** < 0.001**
2005 to 2010	51.43 to 39.50	− 3.4 (− 6.9 to 0.2)	0.100
2010 to 2018	39.50 to 18.59	− 9.6 (− 11.2 to − 8.0)	** < 0.001**
Western provinces			
1997 to 2002	8.61 to 9.83	2.4 (− 6.0 to 11.5)	0.600
2002 to 2005	9.83 to 44.54	52.1 (16.0 to 99.4)	** < 0.001**
2005 to 2010	44.54 to 36.55	− 3.0 (− 9.1 to 3.4)	0.300
2010 to 2018	36.55 to 19.06	− 9.8 (− 11.3 to − 8.3)	** < 0.001**

The bold indicated that the changes were significant

*The number of newly smear-positive TB cases (per 100 000) at the first and the last year of indicated segment

### Changes in newly notified TB cases in different provinces

**Fig. 1:**
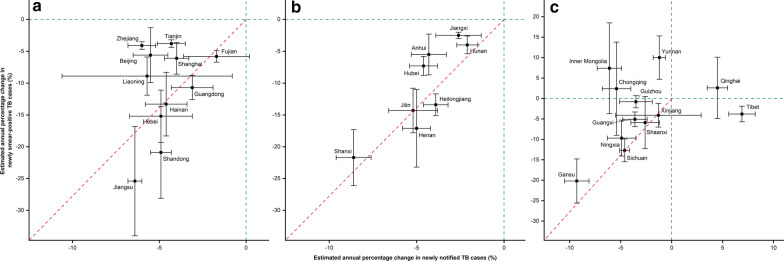
Relationship of recent annual percentage changes in newly smear-positive TB cases with newly notified TB cases in eastern (**a**), central (**b**), and western (**c**) provinces of China^*^. *Annual percentage changes were derived from the last joinpoint of the joinpoint analysis. Horizontal and vertical error bars indicate 95% *CI*s. Changes were significant when the error bars did not cross dashed green lines (0%). The diagonal lines indicate proportional changes of smear-positive and newly notified rates

Ten of the 11 provinces in the eastern region had significant declines in newly notified TB cases over the most recent period (Fig. 1a). The rate of decline ranged from − 3.1% (95% *CI* − 4.3 to − 1.8) for 2005–2018 in Guangdong to − 6.4% (95% *CI* − 6.8 to − 6.1) for 2005–2018 in Jiangsu. Only declining trend was shown for Shanghai (− 4.0%, 95% *CI* − 4.7 to − 3.2) over the period of investigation, without joinpoints. All eight central region provinces also had significant declines in newly notified TB cases over the most recent periods (Fig. 1b). This ranged from − 2.1% (95% *CI* − 2.7 to − 1.4) in Hunan (2005–2018) to − 8.6% (95% *CI* − 9.6 to − 7.5) in Shanxi (2011–2018). The trends of newly notified TB cases in western region provinces have various directions (Fig. 1c). Of the 12 provinces included in western region, nine provinces (Chongqing, Gansu, Guangxi, Guizhou, Inner Mongolia, Ningxia, Shaanxi, Sichuan and Yunnan) had significant declines of newly notified TB cases, two provinces (Qinghai and Tibet) had significant increases, and in the remaining Xinjiang province the annual change of the rate was not significant after the last joinpoint if a joinpoint was identified. Thus, the proportionate changes of newly smear-positive and newly notified cases of TB were different between geographic regions; the eastern and central provinces had more rapid declines in newly smear-positive TB than in newly notified TB (Additional files 1, 2, 3, 4).

### Spatial clustering of annual percentage change in newly notified TB cases

**Fig. 2:**
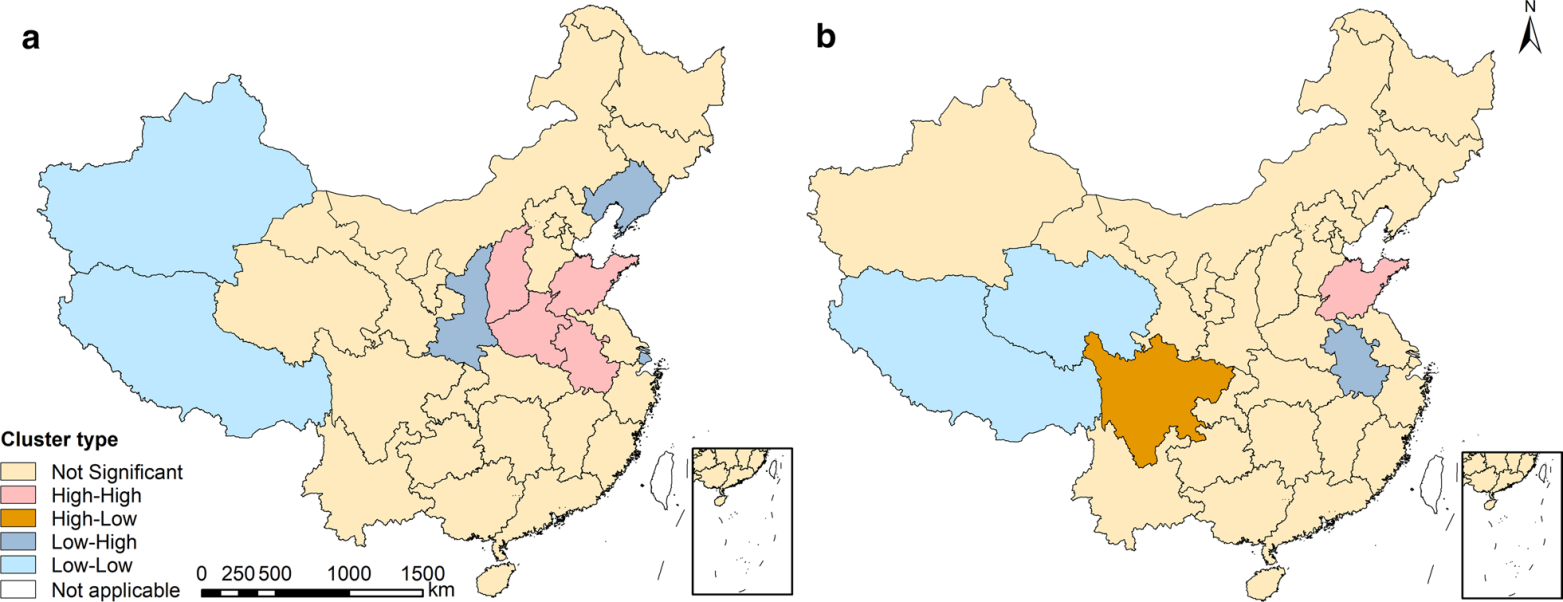
LISA cluster of annual percentage changes in newly notified TB cases (**a**) and newly smear-positive TB cases (**b**) in China

The global Moran’s *I* value for annual percentage change of newly notified TB cases during the most recent period was 0.79 (*P* < 0.05), indicating a significantly positive global spatial auto-correlation. However, the global Moran’s *I* value for annual percentage change of newly smear-positive TB was 0.07 (*P* > 0.1), indicating the global spatial distribution of annual percentage change of newly smear-positive TB was in random mode. The spatial association map for annual percentage change of newly notified TB cases indicated high-high (HH) clustering in Shandong, Henan, Shanxi and Anhui (Fig. 2a). In contrast, there was a low–high (LH) clustering in Shaanxi, Liaoning and Shanghai. This analysis also showed a core “cold spot” cluster of low-low (LL) districts in Xinjiang and Tibet.

Figure 2b, which shows the same analysis for annual percentage change of newly smear-positive TB cases, indicates the distribution of annual percentage change was not significant within most provinces. In particular, Tibet and Qinghai had LL relationships, indicating a low annual percentage change and the surrounding provinces also had low annual percentage changes. Shandong (northern China) had a HH relationship (high annual percentage change), and the surrounding provinces also had high annual percentage changes. On the other hand, Sichuan and Anhui had HL and LH relationships, respectively.

### Regional disparity in the rate of newly notified TB cases

**Fig. 3:**
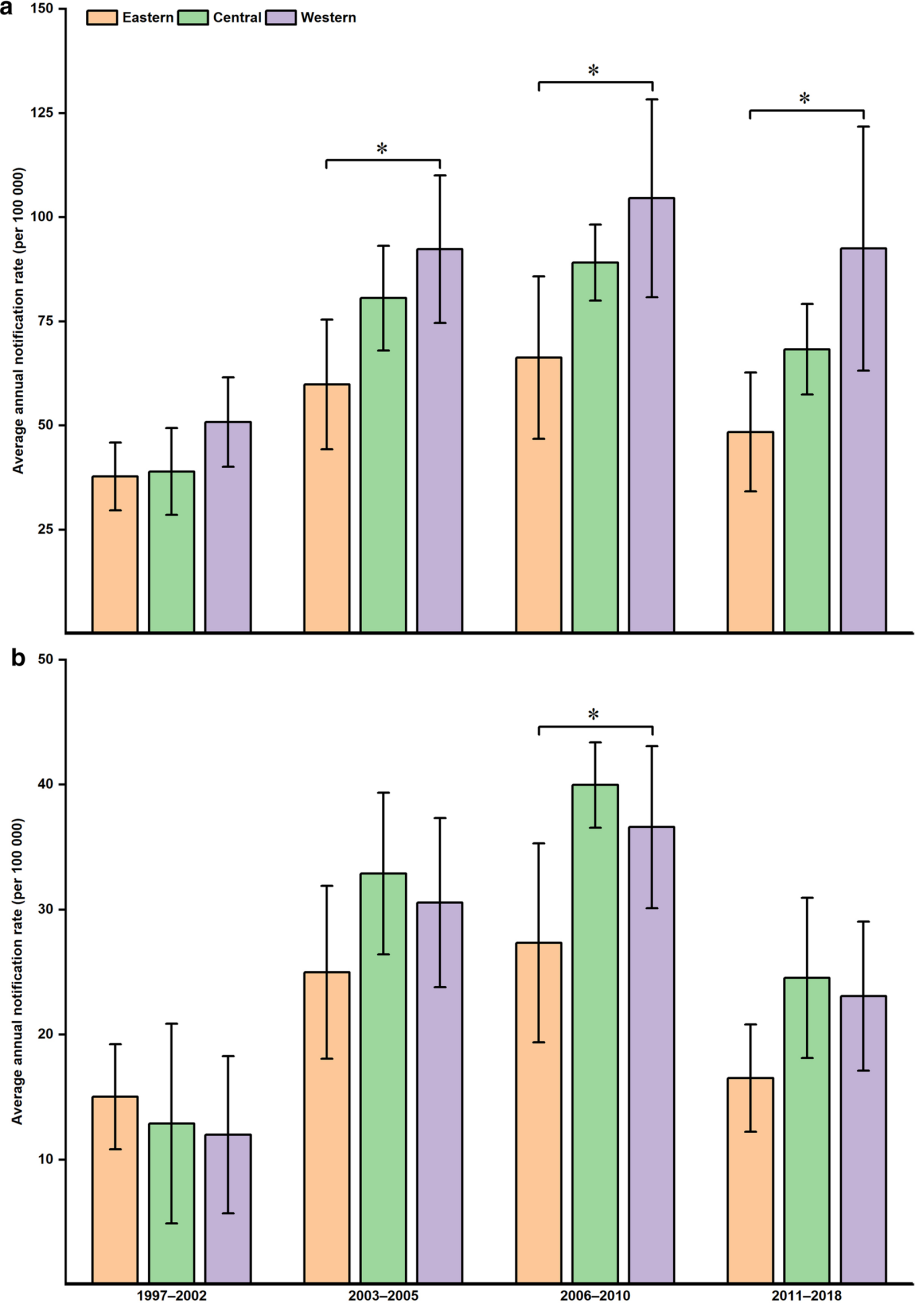
Comparison of newly notified TB cases (**a**) and newly smear-positive TB cases (**b**) in different geographic regions of China during different time periods. *Statistically significant difference (*P* < 0.05). Data are indicated as means and 95% *CI*s

Overall, the average numbers of newly notified and newly smear-positive TB cases per 100 000 varied greatly among the different geographic regions of China. The western region had the highest overall average annual number of newly notified cases (104.53 cases per 100 000) from 2006 to 2010. The central region had highest overall average number of newly smear-positive cases (39.97 cases per 100 000) from 2006 to 2010. Among the four periods identified by joinpoint analysis, those with most newly notified cases per 100 000 were mainly in the western region (Fig. 3a), and those with most newly smear-positive cases per 100 000 were mainly in the central region (Fig. 3b). There were significant geographic differences during 2003–2005, 2006–2010, and 2011–2018 for newly notified TB cases, and during 2006–2010 for newly smear-positive TB cases.

## Discussion

The analysis presented here provides an update of per capita changes in newly notified and smear-positive TB cases from 1997 to 2018 in China. Both of these groups had the greatest numbers of cases during 2005. This was followed by a decrease of 3.8% per year for newly notified cases; the decline of newly smear-positive TB cases was not significant until 2010. Several factors could account for the peak of TB notifications in 2005. First, from 2001 to 2005, China scaled up the DOTS program nationwide and provided free standard short-course chemotherapy in the local CDC system [24]. This led to a great increase in the detection of TB cases. Second, there has been an acceleration of TB control efforts by the government. In particular, China used a series of public health interventions to increase the TB case detection rate after the SARS epidemic [25]. The first of the key interventions that have been implemented was the increased political commitment on combating TB. In March, 2004, the Chinese government made a commitment to achieve the WHO’s 2005 global targets for TB control [26], the Ministry of Health identified 12 provinces with more than 85% of the “missing” cases needed to reach the 70% target for case detection. In addition to increased commitment, the government revised the law on the control of infectious diseases in 2004 [27], this directly benefited TB control by addressing the under-reporting of TB by health facilities. Since TB reporting in China is mandatory, and the TB patients must be referred from the hospital system to the CDC system for regulatory treatment. For referred patients with TB who did not receive treatment, the CDC routinely performs active follow-ups [28]. These improved TB control measures mean that the CDC has been notified of most TB patients, and by the end of 2005 an estimated 80% of the total number of newly smear-positive TB cases received diagnosis and treatment [25]. The rate of newly notified TB cases for China overall also reached the point of inflection in 2005 [29].

In most provinces, we observed declines in the notification rates of new TB cases during recent years. This decline probably reflects a true decrease in TB incidence because almost all provinces in China have had robust web-based surveillance systems since 2003, in line with WHO criteria for TB surveillance and the vital registration system [30]. Even though the annual change of smear-positive TB cases in China overall was not significant from 2005 to 2010, the eastern region had a significant decline. This decrease can be attributed to effective and intensified public health interventions and the good performance of the TB control program. TB control has been improved by increased public-health expenditures, use of an electronic surveillance system, and improved public-health facilities. Moreover, there have been overall improvements in health and well-being in China due to its rapid economic growth.

Our comparison of the rates of change in newly notified and newly smear-positive TB cases indicated a greater increase of smear-positive cases from 2002 to 2005. This can be explained by the commitment of the government to achieve the 2005 global TB control targets of detecting at least 70% of all estimated newly smear-positive TB cases [31, 32]. Similarly, after 2010, the rate of decline of newly smear-positive TB was faster than that of newly notified TB, which indicates a continuing decline in TB transmission.

**Fig. 4:**
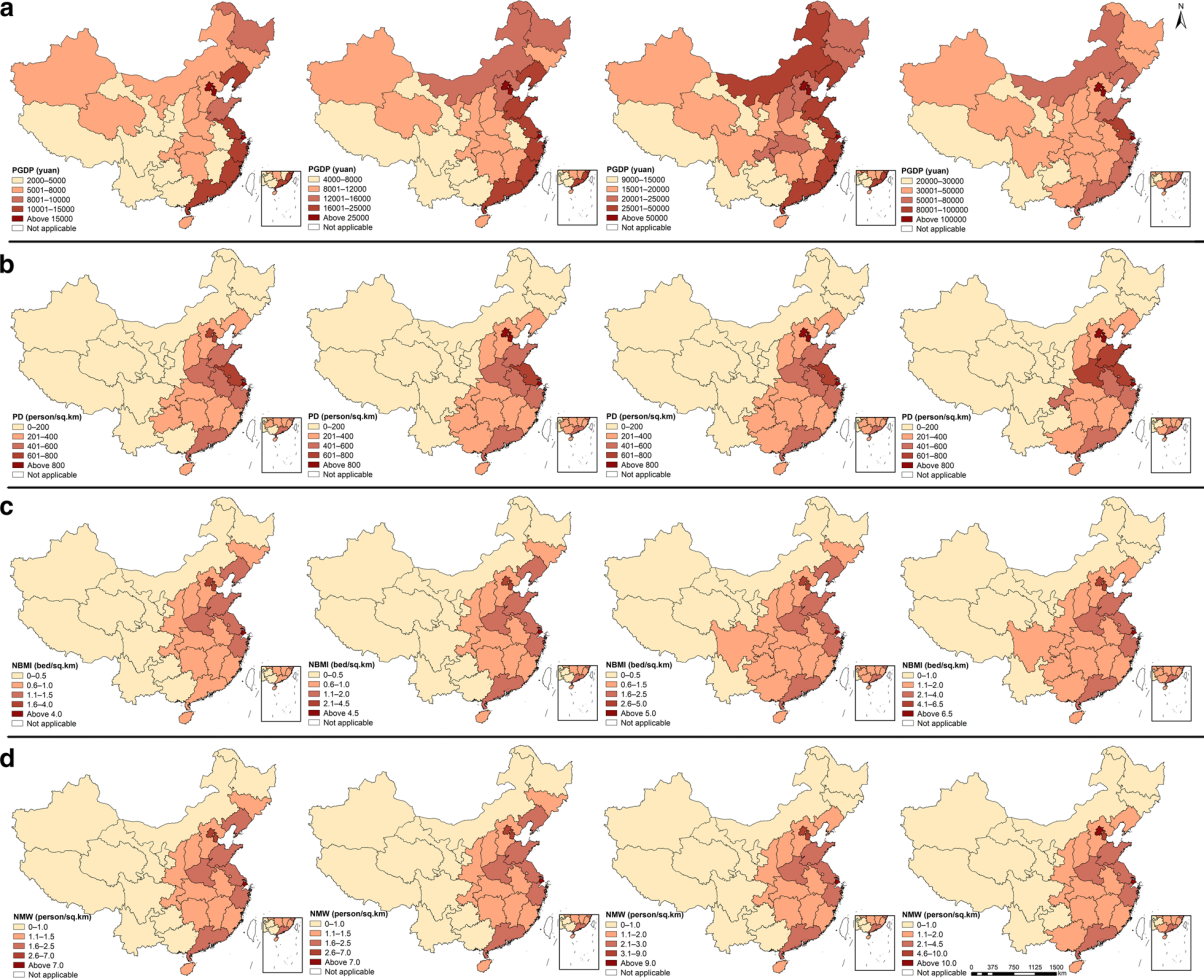
The annual average values of gross domestic product per capita (**a**), population density (**b**), number of beds in medical institutions (**c**) and number of medical personnel (**d**) at province level in China, 1997–2002, 2003–2005, 2006–2010 and 2011–2018

Although the TB notification rate has declined significantly during recent years in China overall, there are large regional disparities. Our findings showed clear regional disparities in annual percentage changes among the provinces. The decline of newly notified TB cases was faster in the eastern and central provinces than in the western provinces. For example, there was a significant decline for Shanghai throughout the whole period of our analysis, but the rate of newly notified cases in Xinjiang remained stable, even after DOTS-based TB control program had been in place for longer than 10 years. Although the rate of newly smear-positive TB decreased in Xinjiang during the most recent years, the decline was about half of that observed during the same period in China overall (− 4.1% vs − 9.9%). In addition to disparities in annual percentage changes, the rate of newly notified TB cases was higher in the western region than in the eastern and central regions. National surveys also showed that the prevalence of bacteriologically confirmed TB among adults in western China was 1.7-times higher than that in the central region and 3.2-times higher than that in the eastern region [8, 33]. These disparities reflect differences in economic development [34]. As shown in Fig. 4, regions with slower economic development tend to have less money, fewer qualified health-care workers, and weaker health facilities, factors known to be associated with high TB burden [35–37]. Additionally, poverty, another risk factor for TB, is more widespread in the underdeveloped western regions of China than in other regions [38]. We suggest increased governmental focus on addressing these regional disparities in TB, especially on the western provinces of China.

This study had some limitations. Firstly, we analyzed routine TB surveillance data, and these data were from annual TB reports of China, so that demographic information, such as sex, age, and occupation, is not available. Secondly, based on data from the reporting system, the notification rates, which are affected by detection ability, could be underestimated. We did not account for underreporting or uncertainty in the data analysis, and this could have potentially introduced bias which limits our interpretation of changes in the notification rate of TB in China. Thirdly, the overall notification rate of TB was affected by the constituent rate in each province; thus, overall notification rate should be considered with reference to each province. Fourth, potential bias could affect reporting of smear-positive TB notifications because of variations in diagnostic standards, technical expertise, and experimental conditions in different departments or institutions. Finally, due to the unavailability of lower administrative levels data, which only provided aggregated data at provincial level, we are not able to stratify the data between urban and rural areas in each province. Given the high population size of many Chinese provinces relative to many other countries, there can be masking of internal contrasts within the province in particular of urban–rural differences.

## Conclusions

In conclusion, although the rates of newly notified and smear-positive TB cases were increasing in China up to about 2005, there was trend for a decrease in 2005, and a strong decrease after 2010. In addition, regional disparities of the TB burden in China should be addressed, particularly the high burden of TB in impoverished regions of the western provinces. These efforts should include sustained efforts to actively find new TB cases, a more effective multi-disciplinary approach to address social determinants that are associated with TB, and ultimately new interventions and techniques that facilitate the early diagnosis and treatment of TB.

## Supplementary Information

**Additional file 1: Table 1.** Joinpoint analysis of newly notified TB cases in each province of China.

**Additional file 2: Table 2.** Joinpoint analysis of newly smear-positive TB cases in each province of China.

**Additional file 3: Table 3.** The test statistic and joinpoint of the best fit model.

**Additional file 4: Table 4.** Estimated Joinpoints of the best fit model.

## Data Availability

The datasets used and analysed during the current study are available from the corresponding author on reasonable request.

## Abbreviations

UNHLM: United Nations High Level Meeting
DOTS: Directly observed treatment, short-course
PGDP: Gross domestic product per capita
PD: Population density
NBMI: Number of beds in medical institutions
NMW: Number of medical personnel
WHO: World Health Organization
BIC: Bayesian information criterion
LISA: Local indicators of spatial association
HH: High-high
HL: High–low
LH: Low–high
LL: Low-low
CDC: Center for Disease Control
